# Antidiabetic effect of *Euterpe oleracea* Mart. (açaí) extract and exercise training on high-fat diet and streptozotocin-induced diabetic rats: A positive interaction

**DOI:** 10.1371/journal.pone.0199207

**Published:** 2018-06-19

**Authors:** Graziele Freitas de Bem, Cristiane Aguiar Costa, Izabelle Barcellos Santos, Viviane da Silva Cristino Cordeiro, Lenize Costa Reis Marins de Carvalho, Marcelo Augusto Vieira de Souza, Ricardo de Andrade Soares, Pergentino José da Cunha Sousa, Dayane Teixeira Ognibene, Angela Castro Resende, Roberto Soares de Moura

**Affiliations:** 1 Department of Pharmacology, Institute of Biology, Rio de Janeiro State University, Rio de Janeiro, RJ, Brazil; 2 Department of Chemical Processes, Institute of Chemistry, Rio de Janeiro State University, Rio de Janeiro, RJ, Brazil; 3 Department of Pharmacy, Federal University of Pará, Belém, PA, Brazil; University of PECS Medical School, HUNGARY

## Abstract

A growing body of evidence suggests a protective role of polyphenols and exercise training on the disorders of type 2 diabetes mellitus (T2DM). We aimed to assess the effect of the açaí seed extract (ASE) associated with exercise training on diabetic complications induced by high-fat (HF) diet plus streptozotocin (STZ) in rats. Type 2 diabetes was induced by feeding rats with HF diet (55% fat) for 5 weeks and a single dose of STZ (35 mg/kg i.p.). Control (C) and Diabetic (D) animals were subdivided into four groups each: Sedentary, Training, ASE Sedentary, and ASE Training. ASE (200 mg/kg/day) was administered by gavage and the exercise training was performed on a treadmill (30min/day; 5 days/week) for 4 weeks after the diabetes induction. In type 2 diabetic rats, the treatment with ASE reduced blood glucose, insulin resistance, leptin and IL-6 levels, lipid profile, and vascular dysfunction. ASE increased the expression of insulin signaling proteins in skeletal muscle and adipose tissue and plasma GLP-1 levels. ASE associated with exercise training potentiated the reduction of glycemia by decreasing TNF-α levels, increasing pAKT and adiponectin expressions in adipose tissue, and IR and pAMPK expressions in skeletal muscle of type 2 diabetic rats. In conclusion, ASE treatment has an antidiabetic effect in type 2 diabetic rats by activating the insulin-signaling pathway in muscle and adipose tissue, increasing GLP-1 levels, and an anti-inflammatory action. Exercise training potentiates the glucose-lowering effect of ASE by activating adiponectin-AMPK pathway and increasing IR expression.

## Introduction

The type 2 diabetes mellitus (T2DM) is one of the most prevalent diseases in the world, being responsible for a marked rate of mortality and morbidity in the general population [1]. The International Diabetes Federation estimated that 415 million adults have diabetes and projected that in 2040 this number will rise to 642 million people [2].

T2DM is a chronic, systemic metabolic disease that is related to a variety of genetic and environmental factors, characterized by elevations in plasma glucose [1]. Insulin resistance precedes and predicts the onset of T2DM in susceptible humans, and contributes to multiple tissue defects characteristic of T2DM, including reduced insulin-stimulated glucose uptake in insulin-sensitive tissues, increased hepatic glucose production, increased lipolysis in adipose tissue, and altered insulin secretion [3,4]. In diabetic patients, the insulin resistance increases the risk of developing serious cardiovascular and metabolic complications [5].

Polyphenols derived from fruits and vegetables are key mediators of antidiabetic effects [6]. They can modulate the digestion of starch and other carbohydrates [7] and, induce satiety [8], mitigate non-enzymatic glycation, modulate hormonal responses [9], among several other effects, which are altogether antidiabetic actions. The plant *Euterpe oleracea* Mart. (*Aracaceae* family) is widely found in the Amazon region of Brazil, and its fruits, popularly known as “açaí” are rich in polyphenols [10].

We have reported that the açaí seed extract (ASE), rich in catechin, epicatechin and polymeric proanthocyanidins [11,12], has an endothelium-dependent vasodilator effect [13], antihypertensive, antioxidant [14–16], and anti-inflammatory properties [11,17], as well as a hypolipidemic effect [12].

In clinical trials, it has been reported the benefits of physical activity and exercise in the prevention of T2DM [18]. Exercise training seems to increase the availability of glucose, and improve the glycemic control and dyslipidemia, reducing the risk of development of cardiovascular disease in patients with T2DM [19].

Until this moment, there is no report on the effect of ASE after the establishment of sustained hyperglycemia in type 2 diabetic rats. Thus, we investigated whether ASE, rich in polyphenols, could reduce hyperglycemia in type 2 diabetic rats and evaluated if a regular exercise training potentiates this effect. In addition, experiments were undertaken to determine the mechanisms involved in the possible antidiabetic effect of ASE associated or not with exercise training in this experimental model.

## Materials and methods

### Preparation of açaí (*Euterpe oleracea* Mart.) seed extract

*Euterpe oleracea* Mart. Fruits were obtained from the Amazon Bay (Pará State, Brazil). The plant was identified at the Goeldi Museum (Belém do Pará, Brazil), where a voucher specimen was deposited under number MG 205222. The hydroalcoholic extract was obtained from the decoction of the açaí seeds, as previously described [13]. Typically, 100 g of seed yielded approximately 5 g of lyophilized extract. The content of polyphenols in ASE, measured by analyzing for total phenol by Folin–Ciocalteu procedure was around 265 mg/g of extract.

### Polyphenol analyses

The analysis of the aqueous fraction residue from ASE by high- performance liquid chromatography (HPLC) and MALDI-TOF mass spectrum was recently reported by our group [11,12]. The HPLC analysis of ASE revealed that it is composed by proanthocyanidins (88% of the total area) and in a minor extent catechin and epicatechin. The chemical and spectrometric analysis revealed that ASE is composed predominantly of polymeric procyanidins, heteropolymers with one gallocatechin unit and, a minor extent, of galloylated procyanidins [12] (S1 File).

### Induction of experimental diabetes in rats

**Fig 1:**
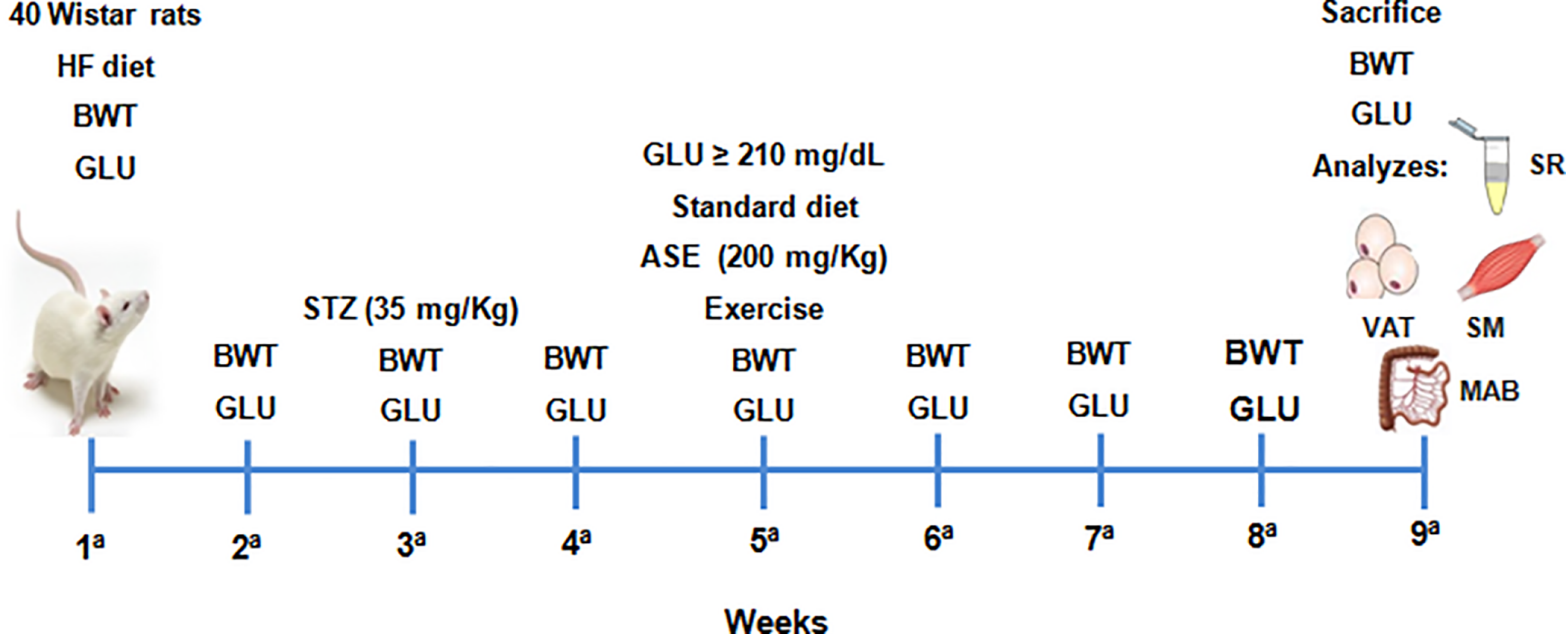
Timeline of the experimental protocol. Schematic figure illustrating the timeline of the experimental protocol. The abbreviations of the figure include HF (high fat); BWT (body weight); GLU (glucose); STZ (streptozotocin); ASE (açaí seed extract); SR (serum); VAT (visceral adipose tissue); SM (skeletal muscle) and MAB (mesenteric arterial bed).

The experiments were conducted in accordance with Brazilian animal protection and welfare laws and the protocol was approved by the Ethics Committee for Experimental Animals Use and Care (CEA) of the Institute of Biology / Rio de Janeiro State University (protocol: CEA/058/2012). The animals were housed in a room with controlled temperature and dark–light cycles. Male Wistar rats weighing 180–200 g were randomly divided into two nutritional groups: a standard chow diet (Control; 10% energy from lipids of soybean oil, 76% from carbohydrate, and 14% from protein; 3.8 kcal/g; n = 40), and a high-fat diet (HF; 55% energy from lipids of pork lard and soybean oil, 31% from carbohydrate, and 14% from protein; 5.2 kcal/g; n = 40). The diets were manufactured by Pragsolutions Biosciences (São Paulo, Brazil), in accordance with the recommendations of the American Institute of Nutrition (AIN-93M) [20]. Three weeks after beginning the experimental diet, HF group was fasted for 12 h (free access to water) and received streptozotocin (STZ) (35 mg/kg in citrate buffer i.p.; pH: 4.4), as previously described [21]. The control group received an intraperitoneal injection of the vehicle solution. Two weeks after STZ injection, and five weeks after beginning the experimental diet, rats from the HF group with blood glucose levels above 11.66 mmol were considered diabetic. At this point, the animals were submitted to exercise training and/or ASE treatment for 4 weeks. ASE (200 mg/kg/day) was administered by intragastric gavage and the exercise training was performed on a treadmill (30 min/day; 5 days/week). During this period, all groups were fed with standard chow diet and glycemia was determined by a glucometer (Accu-Chek Active, Roche, Mannheim, Germany) once a week, as well as body weight (Fig 1). The animals were divided into controls and diabetics treated or not based on their glycemic levels, as previously described [21]. Therefore, this study was performed with eight groups. The Control (C) group was subdivided into: Sedentary C, Training C, ASE Sedentary C and ASE Training C. The Diabetic (D) group was subdivided in: Sedentary D, Training D, ASE Sedentary D and ASE Training D.

### Exercise training

Exercise training was performed on a treadmill (Insight Equipments, Brazil), 5 days per week, 30 minutes per day for 4 weeks. The velocity was progressively increased to 50% to 60% of the maximal velocity obtained during a maximal treadmill stress test, 0% grade [22].

### Insulin, HOMA-IR, HOMA-B and glycosylated hemoglobin (HbA1c)

Insulin concentrations were determined with the Insulin 125I radioimmunoassay (RIA) Kit (MP Biomedicals, LLC-Orangeburg, NY). Homeostasis model assessment of insulin resistance (HOMA-IR), and homeostasis model assessment of β-cell function (HOMA-B) were calculated by the following equations [23]: HOMA-IR = (fasting serum insulin in mU/mL x fasting serum glucose in mg/dL) / (405). HOMA-B = (20 x fasting serum insulin in μIU/mL) / (fasting glucose in mmol/L– 3.5). Determination of serum HbA1c is based on turbidimetric inhibition immunoassay of whole blood hemolysate using a kit (Roche®), performed by automatic analyzer A25 BioSystems®.

### Lipid profile

Serum total cholesterol (TC), high-density lipoprotein (HDL), low-density lipoprotein (LDL) and triglyceride (TG) were analyzed by colorimetric assay (Bioclin, Belo Horizonte, Brazil). The VLDL (very low-density lipoprotein) calculation was done by triglyceride/5.

### Western blotting

The expressions of insulin receptor (IR), protein kinase b (AKT), phosphorylated protein kinase b (pAKT), glucose transporter 4 (GLUT-4) were evaluated in soleus muscle and visceral adipose tissue homogenates. In addition, the expression of adiponectin and phosphorylated adenosine monophosphate-activated protein kinase (pAMPK) were evaluated respectively in adipose tissue and soleus muscle homogenates. Muscle and adipose tissue samples were homogenized in cold lysis buffer (50 mM Tris, pH 7.4, 150 mM NaCl, 0.1% SDS, 5 mM EDTA, 50 mM NaF and 1% Triton X-100) containing Complete Protease Inhibitor Cocktail Tablets (Roche, Basel, Switzerland) using a Ultra-Turrax homogenizer (IKA Werke GmbH & Co. KG, Staufen, Germany). The total protein content was determined by the BCA protein assay kit (Pierce, Rockford, IL, USA). Samples (20 μg total protein) were electrophoresed in 10% tris-glycine sodium dodecyl sulfate-polyacrylamide gels. Proteins were transferred to polyvinylidene fluoride membranes (Hybond ECL; Amersham Pharmacia Biotech, London, UK). The blots were blocked with 5% bovine albumin (Sigma-Aldrich Co., St. Louis, MO, USA) in T-TBS (0.02 M Tris/0.15 M NaCl, pH 7.5, containing 0.1% Tween 20) at room temperature for 1 h and incubated with primary antibodies (1:1000 concentration) overnight at 4°C. After washing with T-TBS, blots were incubated with corresponding secondary conjugated antibodies at 1:5000 and 1:10000 concentration for 1 h. Antibodies were purchased from Santa Cruz Biotechnology Inc. (Santa Cruz, CA). We also incubated all membranes with α-actin antibody to avoid possible inconsistency in protein loading and/or transfer. Blots were developed with enhanced chemiluminescence (ECL; Amersham Biosciences Inc., Piscataway, NJ, USA). The signals were visualized by ChemiDoc Resolutions System and determined by quantitative analysis of digital images of gels using Adobe Photoshop version 13.0 (Adobe System Incorporated).

### Serum assays

The concentrations of interleukin 6 (IL-6), tumor necrosis factor alpha (TNFα), leptin and glucagon-like peptide-1 (GLP-1) were determined by an enzyme-linked immunosorbent assay (ELISA) using commercially available kits (PeproTech®, Rocky Hill, USA; Sigma, St Louis, MO, USA). Briefly, the well plate was pre-coated with capture antibodies (anti-IL-6, anti-TNF-α, anti-leptin and anti-GLP-1) overnight. The plate was washed with phosphate buffer solution and blocked for 1 hour. Then, 100 μl of the samples were added and incubated for 2 h at room temperature. After washing, 100 μl of the respective detection antibody was added and incubated for 2 h at room temperature. After washing, 100 μl of Avidin-horseradish peroxidase conjugate was added and incubated for 30 min at room temperature. Finally, the ABTS liquid substrate was added to the wells and the plate color development was monitored every 5 min for approximately 40 min. The absorbance was measured at 405 nm with wavelength correction set at 650 nm (Bio-Rad Model 550; Hercules, CA, USA). A standard curve was used to determine the amount of IL-6, TNF-α, leptin, and GLP-1 in the samples.

### Vascular perfusion studies

The mesenteric arterial bed (MAB) from the different experimental groups was isolated as previously described [24]. The MAB was rapidly removed, cannulated, and perfused at a flow rate of 4 ml/min with the physiological salt solution (PSS) using a peristaltic pump (Model MINIPLUS 3, Gilson ®). The PSS (composition, mmol/l: NaCl 118, KCl 4.7, CaCl2 2.5, MgSO4 1.2, KH2PO4 1.2, NaHCO3 25, EDTA 0.026, and glucose 6.0) was bubbled with 95% O2 /5% CO2 at 37°C. Perfusion pressure (PP) was measured using a pressure transducer (PowerLab 4/30) and continuously registered on a computer through the LabChart 7 reader program. The drugs were either dissolved in PSS and perfused at the desired concentration or were administered as bolus injections directly into the perfusion stream, close to the arterial cannula. The preparations were left to equilibrate for 30 min. Then, injections of 120 μmol of KCl were administered every 10 mins until consistent responses were obtained. After the equilibration period, basal perfusion pressure (BPP) was elevated (80–100 mm Hg) by adding norepinephrine (NE; 30 μM) to the perfusion solution. After the vasopressor response of NE reached a constant plateau, different doses of acetylcholine (ACh, 1–1000 pmol) and nitroglycerine (NG, 1–1000 nmol) were injected to test the endothelium-dependent and independent vasodilator responses, respectively. NE (1–3000 nmol) was also injected to test the vasoconstrictor responses in MAB with BPP. The vasodilation was expressed as a percentage decrease in the pressor effect of NE and vasoconstriction was represented as a percentage increase in BPP.

### Statistical analysis

Data are shown as the mean and standard error of the mean. Differences among groups were analyzed by a one-way analysis of variance (one-way ANOVA) and post-hoc test of Tukey using IBM SPSS Statistics (IBM corporation software, Inc., Chicago, USA). The Western blotting data were analyzed with the one-way ANOVA and post-hoc test of Tukey for the differences between all groups and the differences between two groups were evaluated by the unpaired Student's t-test using GraphPad Prism version 5.0 (GraphPad Software, San Diego, USA). The glycaemia, body weight and vascular reactivity data were analyzed with the two-way ANOVA and post-hoc test of Tukey using Prism version 5.0 (GraphPad Software, San Diego, USA). P values less than or equal to 0.05 were accepted as statistically significant.

## Results

### Effects of ASE and exercise training on body weight, glycemia, HbA1c and lipid profile

**Fig 2:**
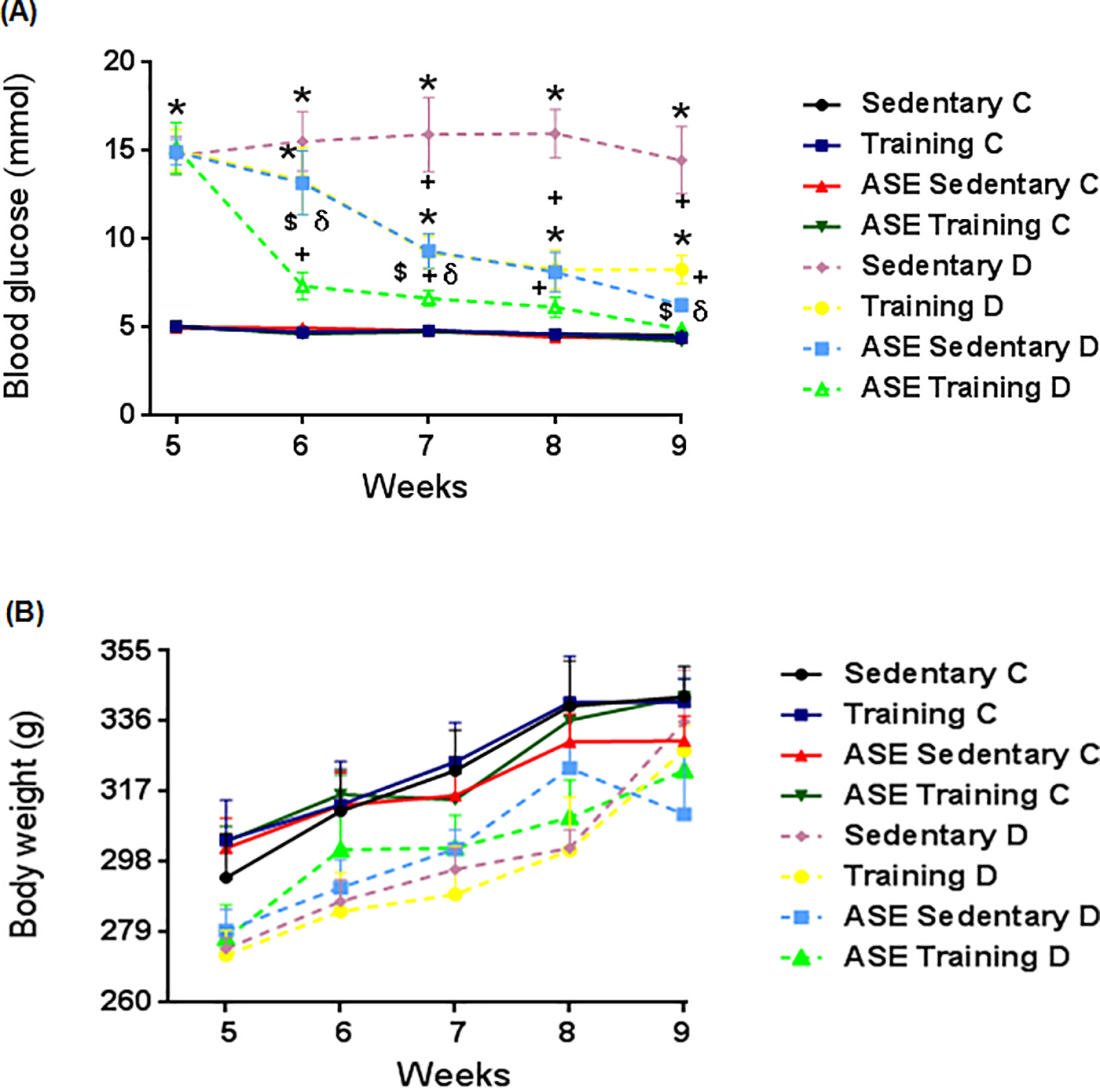
Glycemic levels and body weight. Effect of treatment with ASE (200mg/Kg/day) and exercise training (30 min/day; 5 days per week) on blood glucose levels (A) and body weight (B) in type 2 diabetic rats. Data are mean ± SEM, n = 10 for all groups. *Significantly different (p<0.05) from Controls; ^+^Significantly different (*p ≤ 0*.*05*) from Sedentary D; ^$^Significantly different (*p ≤ 0*.*05*) from Training D; ^δ^Significantly different (*p ≤ 0*.*05*) from ASE Sedentary D.

The body weight was not significantly different among groups during the experimental protocol (Fig 2B), but the body weight gain was different between the weeks (*p ≤ 0*.*05*) in all the groups.

The blood glucose levels were increased (*p ≤ 0*.*05*) after diabetes induction in all type 2 diabetic groups compared with the control groups (Fig 2A). After the first week of treatment, ASE associated with exercise training markedly reduced (*p ≤ 0*.*05*) glycemic levels in type 2 diabetic rats compared with the ASE and/or exercise training alone (Fig 2A).

Final blood glucose levels were elevated (*p ≤ 0*.*05*) in the Sedentary D group compared with the control groups (Fig 2A). Treatment with ASE, or exercise training, reduced (*p ≤ 0*.*05*) the glucose levels in type 2 diabetic rats, compared with the Sedentary D group (Fig 2A). The reduction of glycemia induced by the association of ASE, plus exercise training was significantly higher (*p ≤ 0*.*05*) than the effect of ASE or exercise training alone (Fig 2A). In addition, the association of ASE plus exercise training significantly reduced (*p ≤ 0*.*05*) the blood glucose in ASE Training D rats to levels close to those in the Sedentary C rats (Fig 2A). Also, the glucose levels were different between the weeks (p ≤ 0.05) in all the treated groups.

The Sedentary D group showed an increased (*p ≤ 0*.*05*) HbA1c serum levels compared with the control groups (Table 1), as observed in blood glucose levels. Notably, the treatment with ASE plus exercise training decreased (*p ≤ 0*.*05*) the HbA1c levels compared with Sedentary D and Training D groups (Table 1).

**Table 1:** Effects of ASE (200mg/Kg/day) and exercise training (30 min/day; 5 days per week) on HbA1c serum levels and lipid profile in type 2 diabetic animals.

Variables	Sedentary C	Training C	ASE Sedentary C	ASE Training C	Sedentary D	Training D	ASE Sedentary D	ASE Training D
HbA1c (mg/dL)	3.61 ± 0.06	3.55 ± 0.09	3.66 ± 0.06	3.57 ± 0.05	7.75 ± 0.18*	6.75 ± 0.63*	6.90 ± 0.35*	5.15 ± 0.72*^+^^$^
TG (mg/dL)	46.0 ± 4.81	36.8 ± 2.61	40.2 ± 4.64	31.8 ± 3.49	80.0 ± 3.69*	29.2 ± 4.51^+^	45.0 ± 9.39^+^	41.0 ± 7.22^+^
TC (mg/dL)	38.7 ± 2.51	34.5 ± 1.04	38.9 ± 1.92	34.1 ± 1.10	67.9 ± 6.01*	37.5 ± 2.75^+^	39.9 ± 2.0^+^	32.9 ± 2.54^+^
VLDL (mg/dL)	9.20 ± 0.96	7.36 ± 0.52	8.05 ± 0.93	6.36 ± 0.70	16.0 ± 0.73*	5.85 ± 0.90^+^	9.00 ± 1.87^+^	8.22 ± 1.44^+^
LDL (mg/dL)	7.04 ± 0.93	7.78 ± 1.69	6.88 ± 1.08	7.18 ± 1.52	12.5 ± 1.52	7.18 ± 1.86	7.79 ± 1.63	7.06 ± 1.27
HDL (mg/dL)	33.5 ± 1.92	37.9 ± 2.17	37.8 ± 3.29	39.2 ± 1.14	23.4 ± 1.64*	30.6 ± 2.34	30.9 ± 1.51	34.3 ± 2.06^+^

Data are means ± SEM, n = 6 for all the experiments.

*Significantly different from Controls (*p ≤ 0*.*05*; ANOVA)

^+^Significantly different from Sedentary D (*p ≤ 0*.*05*; ANOVA)

^$^Significantly different from Training D (*p ≤ 0*.*05*; ANOVA).

The serum levels of triglyceride, total cholesterol and VLDL were higher (*p ≤ 0*.*05*) in the Sedentary D group than in the control animals (Table 1). ASE treatment and exercise training, associated or not, reduced (*p ≤ 0*.*05*) those levels in type 2 diabetic rats (Table 1). HDL level was decreased (*p ≤ 0*.*05*) in the Sedentary D group compared with that of control animals (Table 1). Only the ASE treatment associated with exercise training increased the HDL levels (*p ≤ 0*.*05*) in type 2 diabetic groups (Table 1). The LDL levels were not different among groups (Table 1).

### Effects of ASE and exercise training on insulin level, HOMA-IR and HOMA-B

**Fig 3:**
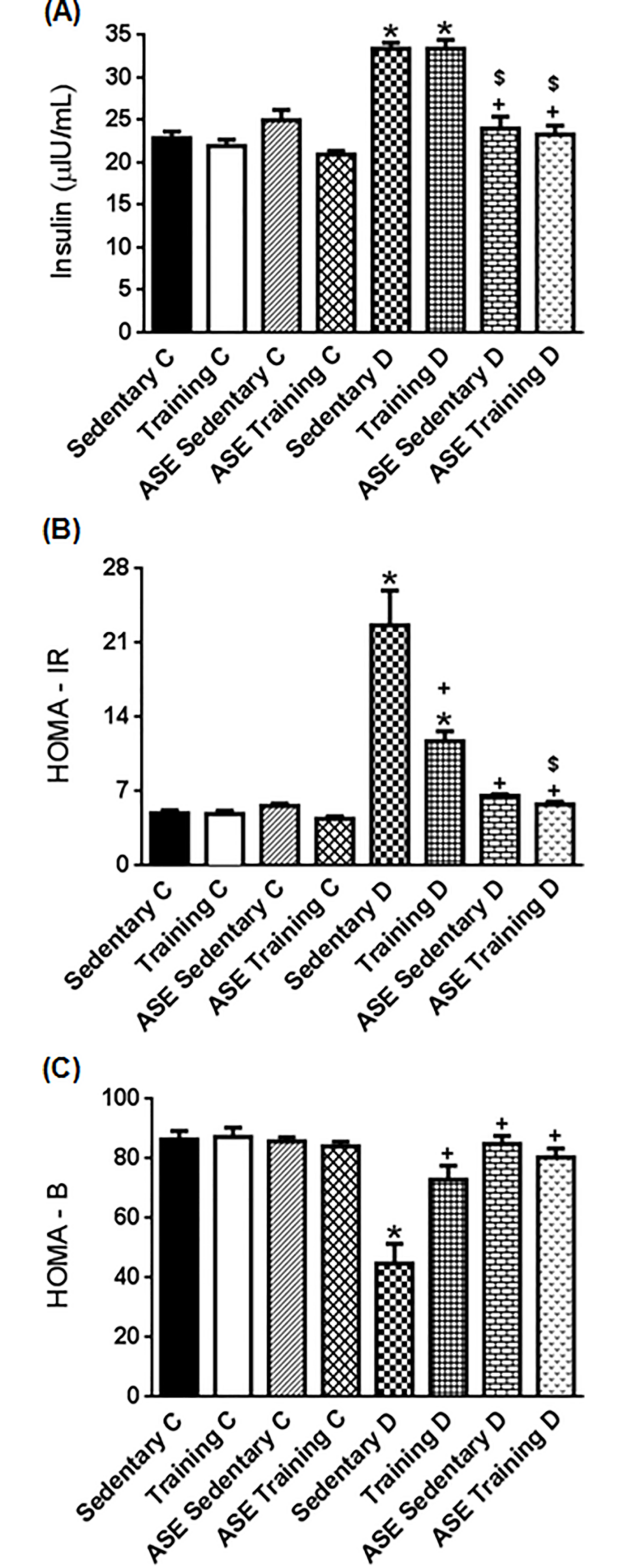
Insulin resistance and β-cell function. Effect of treatment with ASE (200mg/Kg/day) and exercise training (30 min/day; 5 days per week) on serum insulin levels (A), HOMA-IR (B) and HOMA-B (C) indexes in type 2 diabetic rats. Data are mean ± SEM, n = 6 for all groups. *Significantly different (*p ≤ 0*.*05*) from Controls; ^+^Significantly different (*p ≤ 0*.*05*) from Sedentary D. ^$^Significantly different (*p ≤ 0*.*05*) from Training D.

Insulin level was higher (*p ≤ 0*.*05*) in the Sedentary D and Training D animals than in the control groups (Fig 3A). Treatment with ASE, alone or associated with exercise training, reduced (*p ≤ 0*.*05*) the insulin levels (Fig 3A). Exercise training alone did not alter insulin levels in type 2 diabetic rats.

HOMA-IR index was increased (*p ≤ 0*.*05*) in Sedentary D and Training D groups compared with the control groups. Treatment with ASE, as well as exercise training, reduced (*p ≤ 0*.*05*) this index in type 2 diabetic rats compared with the Sedentary D group (Fig 3B). The reduction of insulin resistance induced by the association of ASE, plus exercise training was significantly more pronounced (*p ≤ 0*.*05*) than the effect of exercise training alone (Fig 3B) but was similar to that of the ASE sedentary D group.

HOMA-B index was lower (*p ≤ 0*.*05*) in the Sedentary D group than in control groups (Fig 3C). ASE treatment and exercise training associated or not, increased (*p ≤ 0*.*05*) β-cell function in type 2 diabetic rats (Fig 3C).

### Effects of ASE and exercise training on insulin signaling expression in skeletal muscle

**Fig 4:**
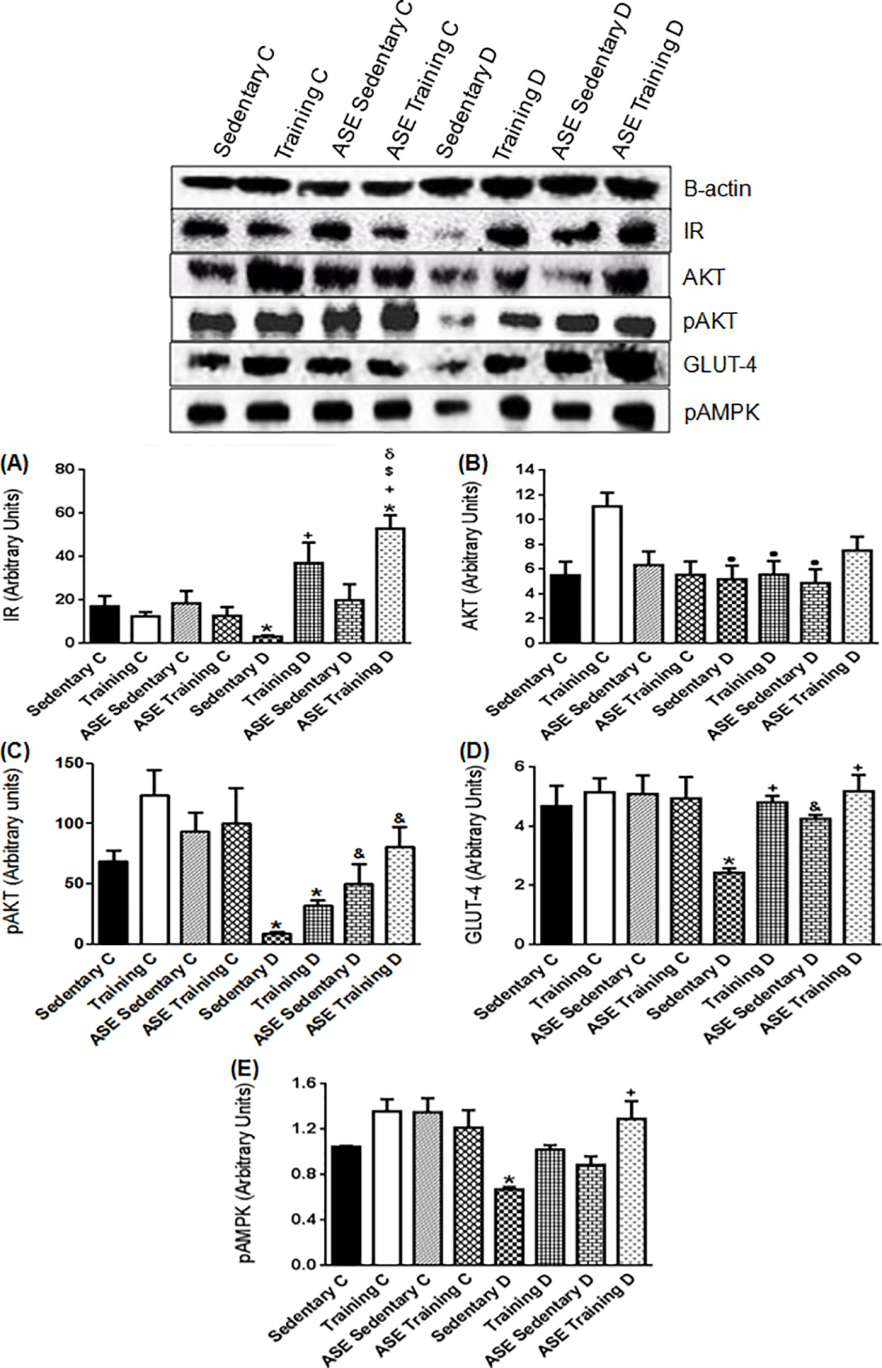
Expression of insulin cascade and pAMPK protein. Effect of treatment with ASE (200mg/Kg/day) and exercise training (30 min/day; 5 days per week) on IR (A), AKT (B), pAKT (C), GLUT-4 (D) and pAMPK expressions in skeletal muscle from type 2 diabetic rats. Data are mean ± SEM, n = 4 for all groups. Using one-way ANOVA: *Significantly different (*p ≤ 0*.*05*) from Controls; ^●^Significantly different (*p ≤ 0*.*05*) from Training C; ^+^Significantly different (*p ≤ 0*.*05*) from Sedentary D; ^$^Significantly different (*p ≤ 0*.*05*) from Training D; ^δ^Significantly different (*p ≤ 0*.*05*) from ASE Sedentary D. Using unpaired Student's t-test: ^&^Significantly different (*p ≤ 0*.*05*) from Sedentary D.

The Sedentary D group showed a decreased (*p ≤ 0*.*05*) IR expression in skeletal muscle compared with the control groups (Fig 4A). The exercise training alone enhanced (*p ≤ 0*.*05*) this protein expression, whereas the ASE treatment alone did not alter the IR expression (Fig 4A). In contrast, the treatment with ASE plus exercise training further increased (*p ≤ 0*.*05*) the IR expression compared with the exercise training alone (Fig 4A).

The expression of AKT was not significantly different between control and diabetic groups, except the Training C group that demonstrated increased (*p ≤ 0*.*05*) AKT content (Fig 4B). In contrast, the expression of pAKT was markedly reduced in the Sedentary D group relative to the control groups (Fig 4C). ASE, but not the exercise training alone increased (*p ≤ 0*.*05*) the pAKT expression in type 2 diabetic rats, without further increase in the ASE training D group (Fig 4C). GLUT-4 expression was lower (*p ≤ 0*.*05*) in the Sedentary D group than in control groups (Fig 4D). The treatment with ASE or exercise training alone or in association increased (*p ≤ 0*.*05*) the expression to levels close to the controls (Fig 4D).

### Effects of ASE and exercise training on pAMPK expression in skeletal muscle

The Sedentary D group showed decreased (*p ≤ 0*.*05*) pAMPK expression in muscle compared with the control groups (Fig 4E). The ASE Training D group presented an increase (*p ≤ 0*.*05*) in this protein content, compared with the Sedentary D (Fig 4E). However, ASE and exercise training alone did not modify the pAMPK expression (Fig 4E).

### Effects of ASE and exercise training on insulin signaling and adiponectin expressions in adipose tissue

**Fig 5:**
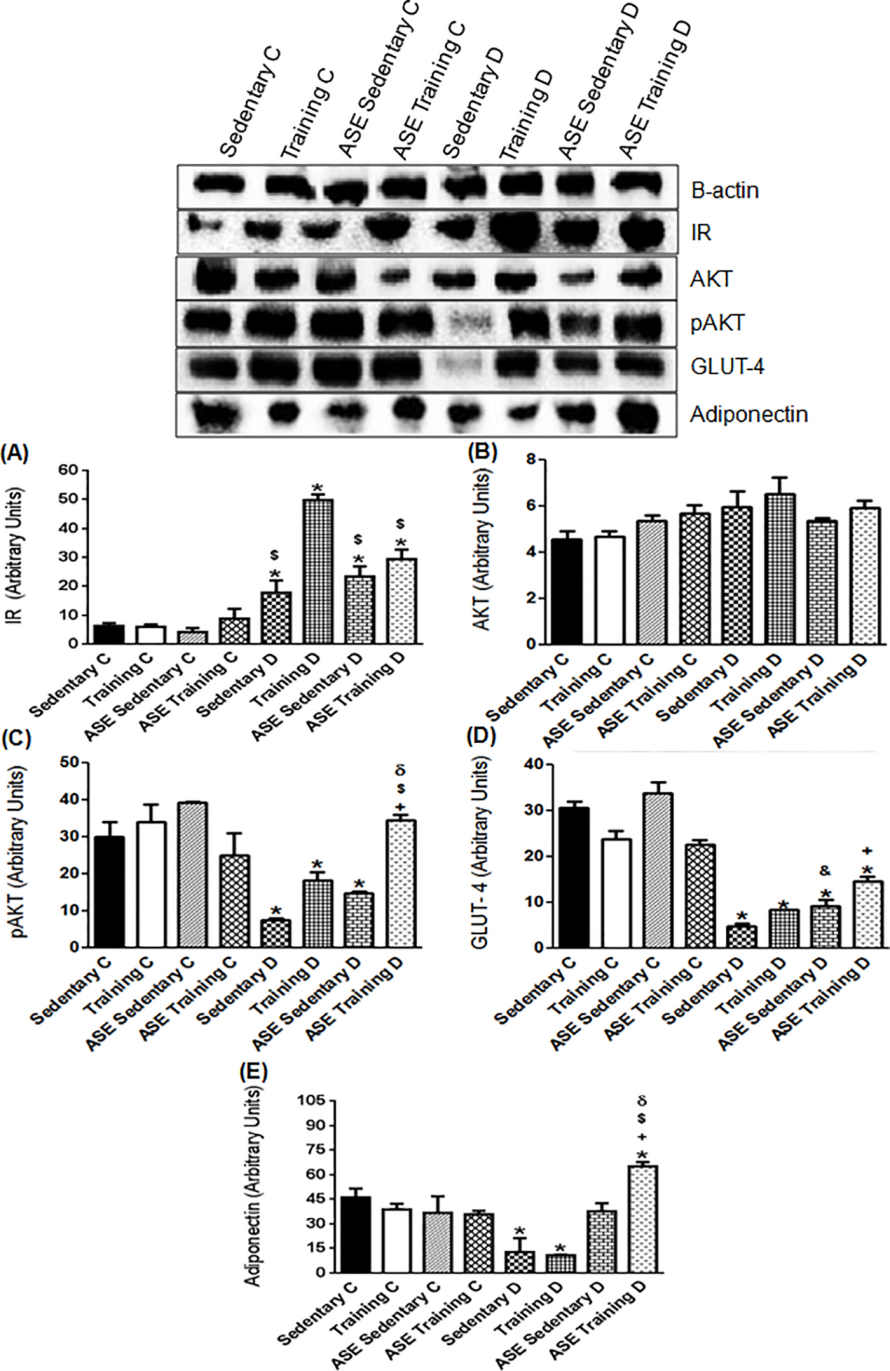
Components of the insulin cascade and adiponectin expression. Effect of treatment with ASE (200mg/Kg/day) and exercise training (30 min/day; 5 days per week) on IR (A), AKT (B), pAKT (C), GLUT-4 (D) and adiponectin (E) expressions in adipose tissue from type 2 diabetic rats. Data are mean ± SEM, n = 4 for all groups. Using one-way ANOVA: *Significantly different (*p ≤ 0*.*05*) from Controls; ^+^Significantly different (p<0.05) from Sedentary D; ^$^Significantly different (*p ≤ 0*.*05*) from Training D; ^δ^Significantly different (*p ≤ 0*.*05*) from ASE Sedentary D. Using unpaired Student's t-test: ^&^Significantly different (*p ≤ 0*.*05*) from Sedentary D.

The expression of IR in adipose tissue was increased (*p ≤ 0*.*05*) in all diabetic groups compared with the control groups (Fig 5A), whereas the exercise training alone increased (*p ≤ 0*.*05*) the expression of this receptor in Training D group compared with all other diabetic groups (Fig 5A).

The expression of AKT was not significantly different between the groups (Fig 5B). However, pAKT expression was reduced in Sedentary D, Training D and ASE Sedentary D groups (*p ≤ 0*.*05*) compared with the control groups (Fig 5C). ASE treatment associated with exercise training markedly increased (*p ≤ 0*.*05*) this protein content in the type 2 diabetic rats (Fig 5C).

All diabetic groups showed decreased (*p ≤ 0*.*05*) expression of GLUT-4 compared with control groups (Fig 5D). However, GLUT-4 content was increased (*p ≤ 0*.*05*) by treatment with ASE alone or associated with exercise training compared with the Sedentary D group (Fig 5D).

Adiponectin protein expression was reduced (*p ≤ 0*.*05*) in Sedentary D and Training D groups compared with control groups (Fig 5E).

The treatment with ASE plus exercise training markedly increased (*p ≤ 0*.*05*) the adiponectin expression (Fig 5E); whereas the tendency of ASE to increase this protein expression was not significantly different (Fig 5E).

### Effects of ASE and exercise training on GLP-1, leptin and proinflammatory cytokines

The Sedentary D group showed a tendency of decreased GLP-1 serum level compared with the control groups (Table 2). The treatment with ASE alone or associated with exercise training increased (*p ≤ 0*.*05*) this incretin level (Table 2). However, exercise training alone did not significantly increase the GLP-1 level in type 2 diabetic rats (Table 2).

**Table 2:** Effects of ASE (200mg/Kg/day) and exercise training (30 min/day; 5 days per week) on glucagon-like peptide-1 (GLP-1), leptin, and anti-inflammatory cytokine serum levels in type 2 diabetic animals.

Variables	Sedentary C	Training C	ASE Sedentary C	ASE Training C	Sedentary D	Training D	ASE Sedentary D	ASE Training D
GLP-1 (pmol/L)	10.1 ± 0.45	8.68 ± 0.61	8.47 ± 0.53	10.0 ± 0.76	6.77 ± 1.44	10.9 ± 0.84	12.4 ± 1.05^+^	11.6 ± 1.0^+^
Leptin (pg/mL)	341.5 ± 50.3	318.0 ± 14.1	373.9 ± 60.6	270.8 ± 10.8	596.0 ± 69*	288.2 ± 50^+^	372.5 ± 28^+^	280.6 ± 19^+^
IL-6 (pg/mL)	22.0 ± 3.6	25.0 ± 4.5	22.7 ± 0.9	22.5 ± 1.7	37.0 ± 2.1*	22.3 ± 1.7^+^	20.2 ± 3.0^+^	20.0 ± 3.0^+^
TNF-α (pg/mL)	60.0 ± 12.6	46.0 ± 10.1	42.4 ± 19.8	70.8 ± 12.5	130 ± 6.04*	74.8 ± 16.7	79.4 ± 17.6	66.8 ± 4.97^+^

Data are means ± SEM, n = 6.

* Significantly different from Controls (*p<0*.*05*; ANOVA)

^+^Significantly different from Sedentary D (*p ≤ 0*.*05*; ANOVA).

The Sedentary D group showed increased (*p ≤ 0*.*05*) leptin serum level compared with the control groups (Table 2). Treatment with ASE, alone or associated with exercise training presented a decrease (*p ≤ 0*.*05*) in this adipokine compared with the Sedentary D (Table 2).

The serum levels of IL-6 and TNF-α were higher (*p ≤ 0*.*05*) in the Sedentary D group than in the control animals (Table 2). ASE treatment and exercise training, associated or not, reduced (*p ≤ 0*.*05*) the IL-6 levels in type 2 diabetic rats (Table 2), whereas only the ASE treatment associated with exercise training reduced (*p ≤ 0*.*05*) the TNF-α levels compared with the Sedentary D group (Table 2).

### Effects of ASE and exercise training on vascular reactivity

**Fig 6:**
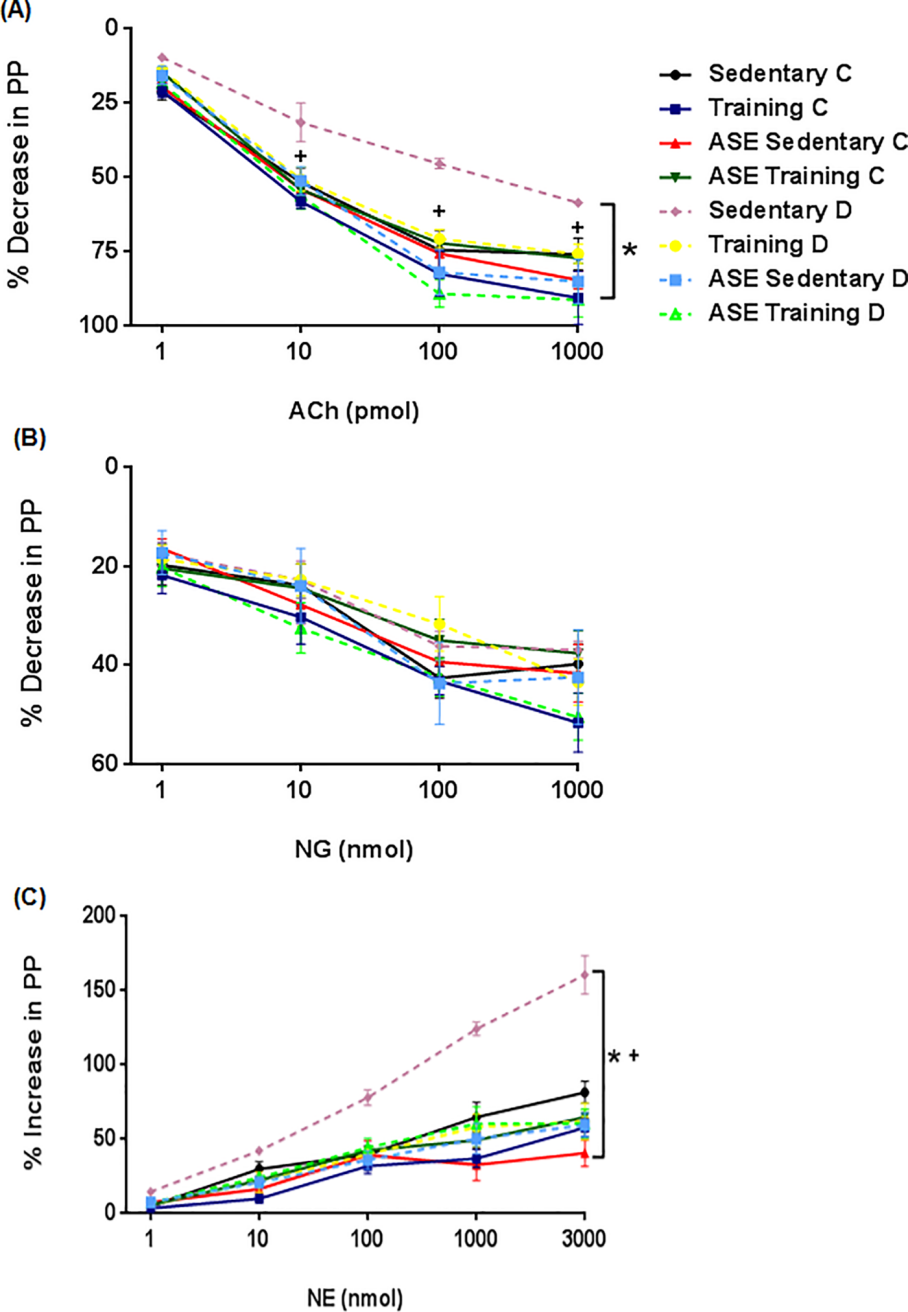
Mesenteric vascular reactivity. Effect of treatment with ASE (200mg/Kg/day) and exercise training (30 min/day; 5 days per week) on vasodilator effects of ACh (A) and NG (B), and vasoconstrictor effects of NE (C) in mesenteric arterial bed from type 2 diabetic rats. Data are mean ± SEM, n = 10 for all groups. *Significantly different (*p ≤ 0*.*05*) from Controls; ^+^Significantly different (*p ≤ 0*.*05*) from Sedentary D.

The reduced (*p ≤ 0*.*05*) ACh-induced vasodilation and the increased (*p ≤ 0*.*05*) NE-induced vasoconstriction in MAB from Sedentary D group were restored (*p ≤ 0*.*05*) by treatment with ASE and exercise training alone or associated in type 2 diabetic rats (Fig 6A and 6C). Endothelium-independent response to NG was not different among groups (Fig 6B). In addition, the vascular responses were different (*p ≤ 0*.*05*) between the doses of ACh, NE, and NG in all the control and diabetic groups, excepted between NG doses in Sedentary D group.

## Discussion

Previous studies in animals have shown that the association of high-fat diet and a low dose of STZ develop many of the features described and observed in human patients with T2DM, suggesting that this model could be used to test antidiabetic agents for the treatment of T2DM [25–27]. Currently, the drugs used in the treatment of diabetes, such as metformin, sulfonylurea, rosiglitazone, and α-glucosidase inhibitors all impart severe side-effects after prolonged treatment [28], demonstrating that the development of new antidiabetic agents is necessary.

Our group has previously demonstrated that treatment with ASE, rich in catechin, epicatechin, and polymeric proanthocyanidins prevented the development of hyperglycemia in obese mice [12] and in type 1 diabetic rats [17]. In the present study, we showed for the first time that treatment with ASE significantly reduced blood glucose levels after diabetes induction. In addition, this effect was more pronounced when ASE was associated with exercise training, and this association was the only one able to reduce glycemic levels in the first week of the treatment and to normalize these levels at the end of the treatment, demonstrating an important antidiabetic effect that improves metabolism and vascular function in type 2 diabetic rats. We also investigated the underlying mechanisms involved in the antidiabetic action of ASE and whether exercise training increases this effect.

In accordance with previous findings [29], we found a significant increase in blood glucose, serum insulin, and HbA1c levels, HOMA index and a significant decrease in HOMA-B index in type 2 diabetic rats. Those metabolic changes were corrected by ASE treatment, except HbA1c levels that were reduced in ASE Training D group, indicating that the antidiabetic effect of ASE is markedly related to the decrease of hyperinsulinemia, reduction in HOMA index and improvement of beta-cell function. These effects may be due to proanthocyanidins and catechins that occur in ASE since those polyphenols decreased intestinal glucose absorption [30,31], have an antioxidant effect that protected beta-cell against toxicity promoted by hyperglycemia [32], and ameliorated insulin sensitivity [33]. However, differently from ASE treatment, a decrease of hyperglycemia induced by exercise training alone was not dependent on the reduction of high insulin levels, which is in agreement with a previous study [34]. This hypoglycemic effect of exercise may be due to the increase of insulin sensitivity and improvement of beta-cell function in type 2 diabetic rats.

The antidiabetic actions observed in the present study may be due to the beneficial effect of ASE on insulin signaling. Skeletal muscle is a key insulin-sensitive organ and plays a major role in maintaining whole-body glucose homeostasis [35]. In T2DM, insulin signaling is disrupted in skeletal muscle, with increased serine phosphorylation of insulin receptor substrate 1 (IRS1), decreased AKT phosphorylation, and reduced translocation of the glucose transporter GLUT4 to the sarcolemmal membrane, thus impairing glucose uptake [3]. These molecular changes were confirmed in the present study in skeletal muscle of Sedentary D group. Our findings also indicate that the reduction of blood glucose levels induced by treatment with ASE may be not due to an increase in IR expression as observed to exercise training, but probably involves upregulation of pAKT and GLUT-4 transporter expressions in muscle. Meanwhile, the hypoglycemic effect induced by exercise training is probably due to the increased expression of IR, suggesting that ASE and exercise training exerts its antidiabetic effects by activation of different points of the insulin signaling pathway in type 2 diabetic rats. Importantly, we also demonstrated that ASE treatment potentiates the effect of exercise training on IR expression, emphasizing a positive correlation between the association and hypoglycemic effect. The insulin-sensitizing effect of polyphenolic compounds in skeletal muscle of diabetic mice was demonstrated by a previous study [36]. It has also been shown that exercise training in type 2 diabetic rats improved insulin-stimulated glucose transport in skeletal muscle [37].

Insulin resistance in adipose tissue of T2DM results in the reduction of glucose uptake and inability to suppress lipolysis, leading to increased release of free fatty acids and glycerol from adipose stores, further increasing plasma free fatty acids [3]. In the current study, we found a lipid dysfunction in the Sedentary D group, which may be induced by insulin resistance with reduced pAKT and GLUT-4 expressions in adipose tissue. Thus, the increase of insulin sensitivity promoted by ASE and exercise training may play an important role in the hypolipidemic effect observed in type 2 diabetic rats. The hypolipidemic effect of ASE was previously described by our group in obese mice [12], and other studies have also shown that polyphenolic compounds activated insulin signaling pathways in skeletal muscle and adipose tissue of diabetic mice and type 2 diabetic rats [36,38].

Interestingly, we observed enhanced IR expression in adipose tissue of all diabetic groups. However, exercise training markedly increased IR expression in the adipose tissue as observed in smooth muscle of type 2 diabetic rats but did not alter downstream insulin signaling proteins, which is not consistent with other studies demonstrating that exercise training up-regulated insulin signaling in adipose tissue of obese rat and type 2 diabetic rats [39,40]. The possible reasons for this divergence may involve the distinct experimental protocols relative to the exercise training. Meanwhile, the improvement in insulin sensitivity promoted by ASE may be at least in part due to an increase in GLUT-4 expression in adipose tissue. Furthermore, ASE treatment associated with exercise training, but not alone, markedly increased the pAKT expression, demonstrating once again a positive correlation of this association. These findings in adipose tissue reinforce the suggestion that ASE and exercise training have different influences on insulin signaling and consequently on the control of glucose metabolism, but when associated may upregulate the insulin pathway as observed for the increased expression of IR in smooth muscle and pAKT in adipose tissue. Altogether, these findings demonstrate an *in vivo* antidiabetic effect, associated with a marked improvement of insulin signaling pathway in muscle and adipose tissues by ASE associated or not with exercise training in type 2 diabetic rats.

Evidence has shown that adiponectin, an adipocyte-secreted adipokine involved in the control of basal metabolism is markedly reduced in T2DM [41], which favors the deregulation in glucose and insulin metabolism [42]. Therefore, the hyperglycemia observed in the Sedentary D group can be mediated in part by the decreased adiponectin protein expression in adipose tissue. The tendency of ASE to increase adiponectin expression is consistent with a previous finding of our group showing increased serum levels of adiponectin in obese mice [12], suggesting that the increase of this adipokine by ASE could play a role in the antidiabetic effect of ASE. We also demonstrated that exercise training alone did not change adiponectin expression in Training D group. However, another study with a different protocol of exercise training demonstrated increased serum levels of adiponectin in type 2 diabetic rats [43]. Notably, ASE treatment associated with exercise training markedly increased the adiponectin expression in type 2 diabetic rats, demonstrating an important role of this association on the antidiabetic effect observed in the present study.

Modulation of the pathophysiology of diabetes induced by adiponectin may be due to the increase in glucose uptake mediated by AMPK activation [44], that stimulate phosphorylation of downstream target of AKT, increasingly, therefore, the translocation of the GLUT-4 transporter [45]. We found in Sedentary D group a reduced skeletal muscle pAMPK expression, which is in agreement with previous findings in the same model [46]. ASE and exercise training alone did not significantly increase pAMPK expression in ASE Sedentary D group, but ASE extract increased this protein expression in liver of type 2 diabetic rats [47]. The metformin, an antidiabetic drug, increased the AMPK activation in the liver after one week of treatment, but not in skeletal muscle, suggesting a time-dependent effect of metformin on the regulation of skeletal muscle AMPK [37], which may also occur in the ASE effect. Notably, ASE treatment associated with exercise training increased pAMPK expression in skeletal muscle of type 2 diabetic rats. Thus, our findings suggest that the activation of the adiponectin-AMPK pathway may play an important role in the early hypoglycemic effect of ASE associated with exercise observed after the first week of the treatment.

Proinflammatory cytokines and high levels of leptin, secreted from the adipose tissue, also contribute to the induction of insulin resistance in T2DM [44]. Consistent with previous findings [48], we found a significant increase in leptin, IL-6 and TNF-α serum levels in type 2 diabetic rats, indicating leptin resistance and inflammation. Notably, ASE and exercise training, alone or associated decreased leptin and IL-6. However, only ASE associated with exercise training reduced TNF-α serum levels in type 2 diabetic rats, which may contribute to the improvement of insulin sensitivity. The polyphenols present in the extract [12] may contribute to the insulin-sensitizing effect of ASE, since it has been reported that ASE, as well as exercise training, decrease leptin serum levels and mediate anti-inflammatory actions [12,17,43,49].

Another important mechanism for glycemic control, GLP-1 an incretin synthesized in the intestine and secreted postprandially, has been reported to stimulate insulin secretion, suppress glucagon secretion and slow gastric emptying [50]. In the present study, the treatment with ASE alone, or associated with exercise training, increased serum GLP-1 levels in type 2 diabetic rats, similar to antidiabetic drugs as exenatide and liraglutide [51]. Considering that ASE is rich in polyphenols [12], our present results are in accordance with a recent study demonstrating that polyphenols from various sources stimulate GLP-1 secretion and increase its half-life by inhibiting dipeptidyl-peptidase-4, an enzyme responsible for its hydrolysis [6]. Therefore, we suggest that the increase in GLP-1 levels also contributes to the antidiabetic effects of ASE. Interesting, we observed that exercise training alone did not alter this incretin, which is in agreement with previous studies [52,53].

Diabetic endothelial dysfunction develops as a consequence of hyperglycemia that disbalances the vasodilator and vasoconstrictor equilibrium, resulting in abnormal vasomotor control [5]. These vascular dysfunctions in type 2 diabetic rats were confirmed in the present study since ACh-induced vasodilation was reduced and NE-induced vasoconstriction was increased in MAB from diabetic animals. ASE and exercise training, alone or in combination reverted these vascular dysfunctions in type 2 diabetic rats. These results are in agreement with previous studies with polyphenolic compounds [54] and exercise training [55]. Previous studies by our group showed that ASE induces vasodilation mediated by nitric oxide/cyclic guanosine monophosphate pathway in combination with endothelium-dependent hyperpolarizing factor [13] and by its antioxidant actions [16, 17]. Meanwhile, the reduced serum levels of the plasminogen activator inhibitor-1 factor, the increased endothelial nitric oxide synthase activity and nitric oxide level, play an important role in the beneficial effect of exercise training relieving the injury degree of endothelial cells and improving the function of fibrinolytic system [56]. These findings suggest that the antidiabetic effect induced by ASE and exercise training alone or in combination, contribute to the improvement of vascular function in type 2 diabetic rats.

## Conclusion

In summary, we demonstrated that ASE treatment has an antidiabetic effect that corrects hyperglycemia, dyslipidemia and vascular dysfunction in type 2 diabetic rats. The mechanisms underlying the antidiabetic effect of ASE may involve the reduction of hyperinsulinemia, activation of insulin-signaling in muscle and adipose tissue, elevation of GLP-1 levels, and an anti-inflammatory property that contribute to the improvement of insulin sensitivity and therefore to glucose-lowering. The beneficial effects of ASE in the type 2 diabetes are increased by exercise training, probably through adiponectin-AMPK pathways and by increasing IR expression in skeletal muscle. This preclinical study opens a possibility for the use of ASE treatment alone or in combination with exercise training in the treatment of T2DM.

## Supporting information

S1 File Chemical composition of ASE. Information about HPLC and MALDI TOF MS analysis of ASE.
(DOC)

## Abbreviations

ACh: acetylcholine
AKT: protein kinase b
ASE: açaí seed extract
C: control
D: diabetic
DM: diabetes mellitus
GLP-1: glucagon-like peptide -1
GLUT-4: glucose transporter
HbA1c: glycosylated hemoglobin
HDL: high-density lipoprotein
HF: high-fat diet
HOMA-B: β-cell function
HOMA-IR: insulin resistance index
IL-6: interleukin—6
IR: insulin receptor
JNK: c-jun n-terminal kinases
LDL: low-density lipoprotein
MABs: mesenteric arterial beds
NE: noradrenaline
NG: nitroglycerin
pAKT: phosphorylated protein kinase b
pAMPK: phosphorylated adenosine monophosphate-activated protein kinase
PKC-Ɵ: protein kinase C theta
SOCS3: suppressor of cytokine signaling 3
STAT 3: signal transducer and activator of transcription
STZ: streptozotocin
T2DM: type 2 diabetes mellitus
TNF-α: tumor necrosis factor alpha
VLDL: very low-density lipoprotein

## References

[1] Chaudhury A, Duvoor C, Reddy Dendi VS, Kraleti S, Chada A, Ravilla R, et al Clinical Review of Antidiabetic Drugs: Implications for Type 2 Diabetes Mellitus Management. Front Endocrinol. 2017; 8: 6 doi: 10.3389/fendo.2017.00006 28167928 10.3389/fendo.2017.00006 PMC5256065

[2] International Diabetes Federation. IDF diabetes atlas Brussels: International Diabetes Federation; 2015.

[3] Gonzalez-Franquesa A, Patti M-E. Insulin Resistance and Mitochondrial Dysfunction. Adv Exp Med Biol. 2017; 982: 465–520. doi: 10.1007/978-3-319-55330-6_25 28551803 10.1007/978-3-319-55330-6_25

[4] Guo C, Huang T, Chen A, Chen X, Wang L, Shen F, et al Glucagon-like peptide 1 improves insulin resistance in vitro through anti-inflammation of macrophages. Braz J Med Biol Res Rev Bras Pesqui Medicas E Biol. 2016; 49: e5826 doi: 10.1590/1414-431X20165826 27878229 10.1590/1414-431X20165826 PMC5188858

[5] Olver TD, Laughlin MH. Endurance, interval sprint, and resistance exercise training: impact on microvascular dysfunction in type 2 diabetes. Am J Physiol Heart Circ Physiol. 2016; 310: H337–350. doi: 10.1152/ajpheart.00440.2015 26408541 10.1152/ajpheart.00440.2015 PMC4796622

[6] Domínguez Avila JA, Rodrigo García J, González Aguilar GA, de la Rosa LA. The Antidiabetic Mechanisms of Polyphenols Related to Increased Glucagon-Like Peptide-1 (GLP1) and Insulin Signaling. Mol Basel Switz. 2017; 22 doi: 10.3390/molecules22060903 28556815 10.3390/molecules22060903 PMC6152752

[7] Nyambe-Silavwe H, Williamson G. Polyphenol- and fibre-rich dried fruits with green tea attenuate starch-derived postprandial blood glucose and insulin: a randomised, controlled, single-blind, cross-over intervention. Br J Nutr. 2016; 116: 443–450. doi: 10.1017/S0007114516002221 27278405 10.1017/S0007114516002221

[8] Campbell CL, Foegeding EA, Harris GK. Cocoa and Whey Protein Differentially Affect Markers of Lipid and Glucose Metabolism and Satiety. J Med Food. 2016; 19: 219–227. doi: 10.1089/jmf.2015.0044 26987021 10.1089/jmf.2015.0044

[9] Grzegorczyk-Karolak I, Gołąb K, Gburek J, Wysokińska H, Matkowski A. Inhibition of Advanced Glycation End-Product Formation and Antioxidant Activity by Extracts and Polyphenols from Scutellaria alpina L. and S. altissima L. Mol Basel Switz. 2016; 21 doi: 10.3390/molecules21060739 27314314 10.3390/molecules21060739 PMC6273165

[10] de Moura RS, Resende ÂC. Cardiovascular and Metabolic Effects of Açaí, an Amazon Plant. J Cardiovasc Pharmacol. 2016; 68: 19–26. doi: 10.1097/FJC.0000000000000347 26657713 10.1097/FJC.0000000000000347

[11] Moura RS de Ferreira TS, Lopes AA Pires KMP, Nesi RT Resende AC, et al Effects of Euterpe oleracea Mart. (AÇAÍ) extract in acute lung inflammation induced by cigarette smoke in the mouse. Phytomedicine Int J Phytother Phytopharm. 2012; 19: 262–269. doi: 10.1016/j.phymed.2011.11.004 22138278 10.1016/j.phymed.2011.11.004

[12] de Oliveira PRB, da Costa CA, de Bem GF, Cordeiro VSC, Santos IB, de Carvalho LCRM, et al Euterpe oleracea Mart.-Derived Polyphenols Protect Mice from Diet-Induced Obesity and Fatty Liver by Regulating Hepatic Lipogenesis and Cholesterol Excretion. PloS One. 2015; 10: e0143721 doi: 10.1371/journal.pone.0143721 26630290 10.1371/journal.pone.0143721 PMC4668108

[13] Rocha APM, Carvalho LCRM, Sousa M a. V, Madeira SVF, Sousa PJC, Tano T, et al Endothelium-dependent vasodilator effect of Euterpe oleracea Mart. (Açaí) extracts in mesenteric vascular bed of the rat. Vascul Pharmacol. 2007; 46: 97–104. doi: 10.1016/j.vph.2006.08.411 17049314 10.1016/j.vph.2006.08.411

[14] Rocha APM, Resende AC, Souza MAV, Carvalho LCRM, Sousa PJC, Tano T, et al Antihypertensive Effects and Antioxidant Action of a Hydro-Alcoholic Extract Obtained from Fruits of Euterpe oleracea Mart. (Acai). J Pharmacol Toxicol. 2008; 3: 435–448. doi: 10.3923/jpt.2008.435.448

[15] da Costa CA, de Oliveira PRB, de Bem GF, de Cavalho LCRM, Ognibene DT, da Silva AFE, et al Euterpe oleracea Mart.-derived polyphenols prevent endothelial dysfunction and vascular structural changes in renovascular hypertensive rats: role of oxidative stress. Naunyn Schmiedebergs Arch Pharmacol. 2012; 385: 1199–1209. doi: 10.1007/s00210-012-0798-z 23052352 10.1007/s00210-012-0798-z

[16] de Bem GF, da Costa CA, de Oliveira PRB, Cordeiro VSC, Santos IB, de Carvalho LCRM, et al Protective effect of Euterpe oleracea Mart (açaí) extract on programmed changes in the adult rat offspring caused by maternal protein restriction during pregnancy. J Pharm Pharmacol. 2014; 66: 1328–1338. doi: 10.1111/jphp.12258 24725099 10.1111/jphp.12258

[17] da Silva Cristino Cordeiro V, de Bem GF, da Costa CA, Santos IB, de Carvalho LCRM, Ognibene DT, et al Euterpe oleracea Mart. seed extract protects against renal injury in diabetic and spontaneously hypertensive rats: role of inflammation and oxidative stress. Eur J Nutr. 2018; 57: 817–832. doi: 10.1007/s00394-016-1371-1 28105508 10.1007/s00394-016-1371-1

[18] Zanuso S, Sacchetti M, Sundberg CJ, Orlando G, Benvenuti P, Balducci S. Exercise in type 2 diabetes: genetic, metabolic and neuromuscular adaptations. A review of the evidence. Br J Sports Med. 2017; doi: 10.1136/bjsports-2016-096724 28501806 10.1136/bjsports-2016-096724

[19] Umpierre D, Ribeiro PAB, Kramer CK, Leitão CB, Zucatti ATN, Azevedo MJ, et al Physical activity advice only or structured exercise training and association with HbA1c levels in type 2 diabetes: a systematic review and meta-analysis. JAMA. 2011; 305: 1790–1799. doi: 10.1001/jama.2011.576 21540423 10.1001/jama.2011.576

[20] Reeves PG, Nielsen FH, Fahey GC. AIN-93 purified diets for laboratory rodents: final report of the American Institute of Nutrition ad hoc writing committee on the reformulation of the AIN-76A rodent diet. J Nutr. 1993; 123: 1939–1951. doi: 10.1093/jn/123.11.1939 8229312 10.1093/jn/123.11.1939

[21] Subhasree N, Kamella A, Kaliappan I, Agrawal A, Dubey GP. Antidiabetic and antihyperlipidemic activities of a novel polyherbal formulation in high fat diet/streptozotocin induced diabetic rat model. 2015; 47: 509–513. doi: 10.4103/0253-7613.165200 26600639 10.4103/0253-7613.165200 PMC4621671

[22] Matsuura C, Brunini TMC, Carvalho LCMM, Resende AC, Carvalho JJ, de Castro JPW, et al Exercise training in doxorubicin-induced heart failure: effects on the L-arginine-NO pathway and vascular reactivity. J Am Soc Hypertens JASH. 2010; 4: 7–13. doi: 10.1016/j.jash.2009.10.005 20374946 10.1016/j.jash.2009.10.005

[23] Matthews DR, Hosker JP, Rudenski AS, Naylor BA, Treacher DF, Turner RC. Homeostasis model assessment: insulin resistance and beta-cell function from fasting plasma glucose and insulin concentrations in man. Diabetologia. 1985; 28: 412–419. 3899825 10.1007/BF00280883

[24] Mcgregor DD. The effect of sympathetic nerve stimulation of vasoconstrictor responses in perfused mesenteric blood vessels of the rat. J Physiol. 1965; 177: 21–30. 14296957 10.1113/jphysiol.1965.sp007572 PMC1357221

[25] Sugano M, Yamato H, Hayashi T, Ochiai H, Kakuchi J, Goto S, et al High-fat diet in low-dose-streptozotocin-treated heminephrectomized rats induces all features of human type 2 diabetic nephropathy: a new rat model of diabetic nephropathy. Nutr Metab Cardiovasc Dis NMCD. 2006; 16: 477–484. doi: 10.1016/j.numecd.2005.08.007 17015185 10.1016/j.numecd.2005.08.007

[26] Xing X-H, Zhang Z-M, Hu X-Z, Wu R-Q, Xu C. Antidiabetic effects of Artemisia sphaerocephala Krasch. gum, a novel food additive in China, on streptozotocin-induced type 2 diabetic rats. J Ethnopharmacol. 2009; 125: 410–416. doi: 10.1016/j.jep.2009.07.021 19635546 10.1016/j.jep.2009.07.021

[27] Gaballah HH, Zakaria SS, Mwafy SE, Tahoon NM, Ebeid AM. Mechanistic insights into the effects of quercetin and/or GLP-1 analogue liraglutide on high-fat diet/streptozotocin-induced type 2 diabetes in rats. Biomed Pharmacother Biomedecine Pharmacother. 2017; 92: 331–339. doi: 10.1016/j.biopha.2017.05.086 28554128 10.1016/j.biopha.2017.05.086

[28] Jiao Y, Wang X, Jiang X, Kong F, Wang S, Yan C. Antidiabetic effects of Morus alba fruit polysaccharides on high-fat diet- and streptozotocin-induced type 2 diabetes in rats. J Ethnopharmacol. 2017; 199: 119–127. doi: 10.1016/j.jep.2017.02.003 28163112 10.1016/j.jep.2017.02.003

[29] Sharma S, Pathak S, Gupta G, Sharma SK, Singh L, Sharma RK, et al Pharmacological evaluation of aqueous extract of syzigium cumini for its antihyperglycemic and antidyslipidemic properties in diabetic rats fed a high cholesterol diet-Role of PPARγ and PPARα. Biomed Pharmacother Biomedecine Pharmacother. 2017; 89: 447–453. doi: 10.1016/j.biopha.2017.02.048 28249245 10.1016/j.biopha.2017.02.048

[30] Shimizu M, Kobayashi Y, Suzuki M, Satsu H, Miyamoto Y. Regulation of intestinal glucose transport by tea catechins. BioFactors Oxf Engl. 2000; 13: 61–65. 10.1002/biof.5520130111 11237201

[31] Schäfer A, Högger P. Oligomeric procyanidins of French maritime pine bark extract (Pycnogenol) effectively inhibit alpha-glucosidase. Diabetes Res Clin Pract. 2007; 77: 41–46. doi: 10.1016/j.diabres.2006.10.011 17098323 10.1016/j.diabres.2006.10.011

[32] Carlessi R, Keane KN, Mamotte C, Newsholme P. Nutrient regulation of β-cell function: what do islet cell/animal studies tell us? Eur J Clin Nutr. 2017; 71: 890–895. doi: 10.1038/ejcn.2017.49 28422118 10.1038/ejcn.2017.49

[33] Babu PVA, Liu D, Gilbert ER. Recent advances in understanding the anti-diabetic actions of dietary flavonoids. J Nutr Biochem. 2013; 24: 1777–1789. doi: 10.1016/j.jnutbio.2013.06.003 24029069 10.1016/j.jnutbio.2013.06.003 PMC3821977

[34] Slentz CA, Tanner CJ, Bateman LA, Durheim MT, Huffman KM, Houmard JA, et al Effects of exercise training intensity on pancreatic beta-cell function. Diabetes Care. 2009; 32: 1807–1811. doi: 10.2337/dc09-0032 19592624 10.2337/dc09-0032 PMC2752909

[35] Al-Khalili L, Krämer D, Wretenberg P, Krook A. Human skeletal muscle cell differentiation is associated with changes in myogenic markers and enhanced insulin-mediated MAPK and PKB phosphorylation. Acta Physiol Scand. 2004; 180: 395–403. doi: 10.1111/j.1365-201X.2004.01259.x 15030381 10.1111/j.1365-201X.2004.01259.x

[36] Soares de Moura R, da Costa GF, Moreira ASB, Queiroz EF, Moreira DDC, Garcia-Souza EP, et al Vitis vinifera L. grape skin extract activates the insulin-signalling cascade and reduces hyperglycaemia in alloxan-induced diabetic mice. J Pharm Pharmacol. 2012; 64: 268–276. doi: 10.1111/j.2042-7158.2011.01395.x 22221103 10.1111/j.2042-7158.2011.01395.x

[37] Smith AC, Mullen KL, Junkin KA, Nickerson J, Chabowski A, Bonen A, et al Metformin and exercise reduce muscle FAT/CD36 and lipid accumulation and blunt the progression of high-fat diet-induced hyperglycemia. Am J Physiol Endocrinol Metab. 2007; 293: E172–181. doi: 10.1152/ajpendo.00677.2006 17374701 10.1152/ajpendo.00677.2006

[38] Cai S, Sun W, Fan Y, Guo X, Xu G, Xu T, et al Effect of mulberry leaf (Folium Mori) on insulin resistance via IRS-1/PI3K/Glut-4 signalling pathway in type 2 diabetes mellitus rats. Pharm Biol. 2016; 54: 2685–2691. doi: 10.1080/13880209.2016.1178779 27158744 10.1080/13880209.2016.1178779

[39] da Luz G, Frederico MJS, da Silva S, Vitto MF, Cesconetto PA, de Pinho RA, et al Endurance exercise training ameliorates insulin resistance and reticulum stress in adipose and hepatic tissue in obese rats. Eur J Appl Physiol. 2011; 111: 2015–2023. doi: 10.1007/s00421-010-1802-2 21249392 10.1007/s00421-010-1802-2

[40] Liang Y, Sheng S, Fang P, Ma Y, Li J, Shi Q, et al Exercise-induced galanin release facilitated GLUT4 translocation in adipocytes of type 2 diabetic rats. Pharmacol Biochem Behav. 2012; 100: 554–559. doi: 10.1016/j.pbb.2011.10.026 22079346 10.1016/j.pbb.2011.10.026

[41] Katsiki N, Mantzoros C, Mikhailidis DP. Adiponectin, lipids and atherosclerosis. Curr Opin Lipidol. 2017; 28: 347–354. doi: 10.1097/MOL.0000000000000431 28463859 10.1097/MOL.0000000000000431

[42] de Las Heras N, Valero-Muñoz M, Martín-Fernández B, Ballesteros S, López-Farré A, Ruiz-Roso B, et al Molecular factors involved in the hypolipidemic- and insulin-sensitizing effects of a ginger (Zingiber officinale Roscoe) extract in rats fed a high-fat diet. Appl Physiol Nutr Metab Physiol Appl Nutr Metab. 2017; 42: 209–215. doi: 10.1139/apnm-2016-0374 28125276 10.1139/apnm-2016-0374

[43] Teixeira de Lemos E, Pinto R, Oliveira J, Garrido P, Sereno J, Mascarenhas-Melo F, et al Differential effects of acute (extenuating) and chronic (training) exercise on inflammation and oxidative stress status in an animal model of type 2 diabetes mellitus. Mediators Inflamm. 2011; 2011: 253061 doi: 10.1155/2011/253061 22174491 10.1155/2011/253061 PMC3235883

[44] Choe SS, Huh JY, Hwang IJ, Kim JI, Kim JB. Adipose Tissue Remodeling: Its Role in Energy Metabolism and Metabolic Disorders. Front Endocrinol. 2016; 7: 30 doi: 10.3389/fendo.2016.00030 27148161 10.3389/fendo.2016.00030 PMC4829583

[45] Viollet B, Andreelli F. AMP-activated protein kinase and metabolic control. Handb Exp Pharmacol. 2011; 303–330. doi: 10.1007/978-3-642-17214-4_13 21484577 10.1007/978-3-642-17214-4_13 PMC3384586

[46] Guo Z, Qin Z, Zhang R, Li J, Yin Y. Effect of rosiglitazone on the expression of cardiac adiponectin receptors and NADPH oxidase in type 2 diabetic rats. Eur J Pharmacol. 2012; 685: 116–125. doi: 10.1016/j.ejphar.2012.04.010 22542658 10.1016/j.ejphar.2012.04.010

[47] de Bem GF, da Costa CA, da Silva Cristino Cordeiro V, Santos IB, de Carvalho LCRM, de Andrade Soares R, et al Euterpe oleracea Mart. (açaí) seed extract associated with exercise training reduces hepatic steatosis in type 2 diabetic male rats. J Nutr Biochem. 2018; 52: 70–81. doi: 10.1016/j.jnutbio.2017.09.021 29175669 10.1016/j.jnutbio.2017.09.021

[48] Hazman Ö, Ovalı S. Investigation of the anti-inflammatory effects of safranal on high-fat diet and multiple low-dose streptozotocin induced type 2 diabetes rat model. Inflammation. 2015; 38: 1012–1019. doi: 10.1007/s10753-014-0065-1 25411096 10.1007/s10753-014-0065-1

[49] Yi X, Cao S, Chang B, Zhao D, Gao H, Wan Y, et al Effects of acute exercise and chronic exercise on the liver leptin-AMPK-ACC signaling pathway in rats with type 2 diabetes. J Diabetes Res. 2013; 2013: 946432 doi: 10.1155/2013/946432 24455748 10.1155/2013/946432 PMC3877642

[50] Umpierrez GE, Bailey TS, Carcia D, Shaefer C, Shubrook JH, Skolnik N. Improving postprandial hyperglycemia in patients with type 2 diabetes already on basal insulin therapy: a review of current strategies. J Diabetes. 2017; doi: 10.1111/1753-0407.12576 28581207 10.1111/1753-0407.12576

[51] Fineman MS, Cirincione BB, Maggs D, Diamant M. GLP-1 based therapies: differential effects on fasting and postprandial glucose. Diabetes Obes Metab. 2012; 14: 675–688. doi: 10.1111/j.1463-1326.2012.01560.x 22233527 10.1111/j.1463-1326.2012.01560.x

[52] Delghingaro-Augusto V, Décary S, Peyot M-L, Latour MG, Lamontagne J, Paradis-Isler N, et al Voluntary running exercise prevents β-cell failure in susceptible islets of the Zucker diabetic fatty rat. Am J Physiol Endocrinol Metab. 2012; 302: E254–264. doi: 10.1152/ajpendo.00360.2011 22045312 10.1152/ajpendo.00360.2011

[53] Keller AC, Knaub LA, Miller MW, Birdsey N, Klemm DJ, Reusch JEB. Saxagliptin restores vascular mitochondrial exercise response in the Goto-Kakizaki rat. J Cardiovasc Pharmacol. 2015; 65: 137–147. doi: 10.1097/FJC.0000000000000170 25264749 10.1097/FJC.0000000000000170 PMC4320657

[54] Kamata K, Makino A, Kanie N, Oda S, Matsumoto T, Kobayashi T, et al Effects of anthocyanidin derivative (HK-008) on relaxation in rat perfused mesenterial bed. J Smooth Muscle Res Nihon Heikatsukin Gakkai Kikanshi. 2006; 42: 75–88. 17001114 10.1540/jsmr.42.75

[55] Mota MM, Silva TLTB da, Fontes MT, Barreto AS, Araújo JE dos S, Oliveira ACC de, et al Resistance exercise restores endothelial function and reduces blood pressure in type 1 diabetic rats. Arq Bras Cardiol. 2014; 103: 25–32. doi: 10.5935/abc.20140087 25120082 10.5935/abc.20140087 PMC4126758

[56] Chengji W, Xianjin F. Treadmill exercise alleviates diabetic cardiomyopathy by suppressing plasminogen activator inhibitor expression and enhancing eNOS in streptozotocin-induced male diabetic rats. Endocr Connect. 2018; 7: 553–559. doi: 10.1530/EC-18-0060 29555653 10.1530/EC-18-0060 PMC5887130

