# Supplementary material for: Antidiabetic effect of Euterpe oleracea Mart. (açaí) extract and exercise training on high-fat diet and streptozotocin-induced diabetic rats: A positive interaction
Source: PLoS One. 2018 Jun 19;13(6):e0199207. doi: 10.1371/journal.pone.0199207 (PMC6007924; doi:10.1371/journal.pone.0199207)
Supplement: S1 File — Information about HPLC and MALDI TOF MS analysis of ASE. (DOC) [file pone.0199207.s001.doc]

**SUPPLEMENTARY MATERIAL**

**Chemical Composition of ASE**

Fig 1A shows the chromatogram of the water fraction residue. The big peak eluting last (51min) displays UV spectral characteristics of condensed tannins and contains predominantly polymeric condensed tannins, proanthocyanidins. The integrated area of this peak is equivalent to 88% of the total area, showing that the extract is composed basically by proanthocyanidins. The peaks at 27 and 38 min are of catechin and epicatechin, respectively, and were identified after co-injection with reference standards. Direct infusion negative ESI MS analysis of the ethyl acetate fraction residue revealed the presence of catechin, epicatechin, a dimer and a trimer at m/z 289, 577 and 865 respectively.

Further experiments were performed to delineate the composition of the main fraction of ASE, the water residue: acid depolymerization in presence of phloroglucinal led to the determination of the mDP as 6.7 and normal phase chromatography was used to assess the extent of polymerization allowing the detection of tannin monomers to decamers (Fig 1B).

MALDI TOF MS has proved to be highly suited to the analysis of the highly complex proanthocyanidin samples (Monagas M et al., J Pharmaceutical and Biomedical Anal. 2010; 51: 358–372). The MALDI TOF MS spectrum of the water residue sample (Fig 2) has shown two major B-type proanthocyanidin peak sequences: the first one of procyanidins (from trimer to undecamer) at m/z (sodium adducts): 889, 1177, 1465, 1753, 2041, 2329, 2617, 2907 and 3193. The second sequence of signals presented peaks separated by 16 mass units from the previous one indicating heteropolymerization with introduction of one unit of (epi) galocatechin from the trimer to undecamer, peaks at m/z (sodium adducts): 905, 1193, 1481, 1769, 2058, 2347, 2634, 2922 and 3211). 3-O-galloylated series were also detected with procyanidin peaks added by multiples of 152 mass units: monogalloylated trimer, mono, di and trigalloylatedtetramer; mono, di and trigalloylatedpentamer all detected in very small amounts. Application of MALDI TOF MS in this field has been critical to unravel the structural complexities of proanthocyanidin samples (Stringano E et al., Anal Chem. 2011; 83(11): 4147–41).

Thus, the application of chemical and spectrometric methodology to the analysis of the composition of ASE revealed that it is composed predominantly by polymeric procyanidins, heteropolymers with one gallocatechin unit and, in minor extent, of galloylatedprocyanidins.


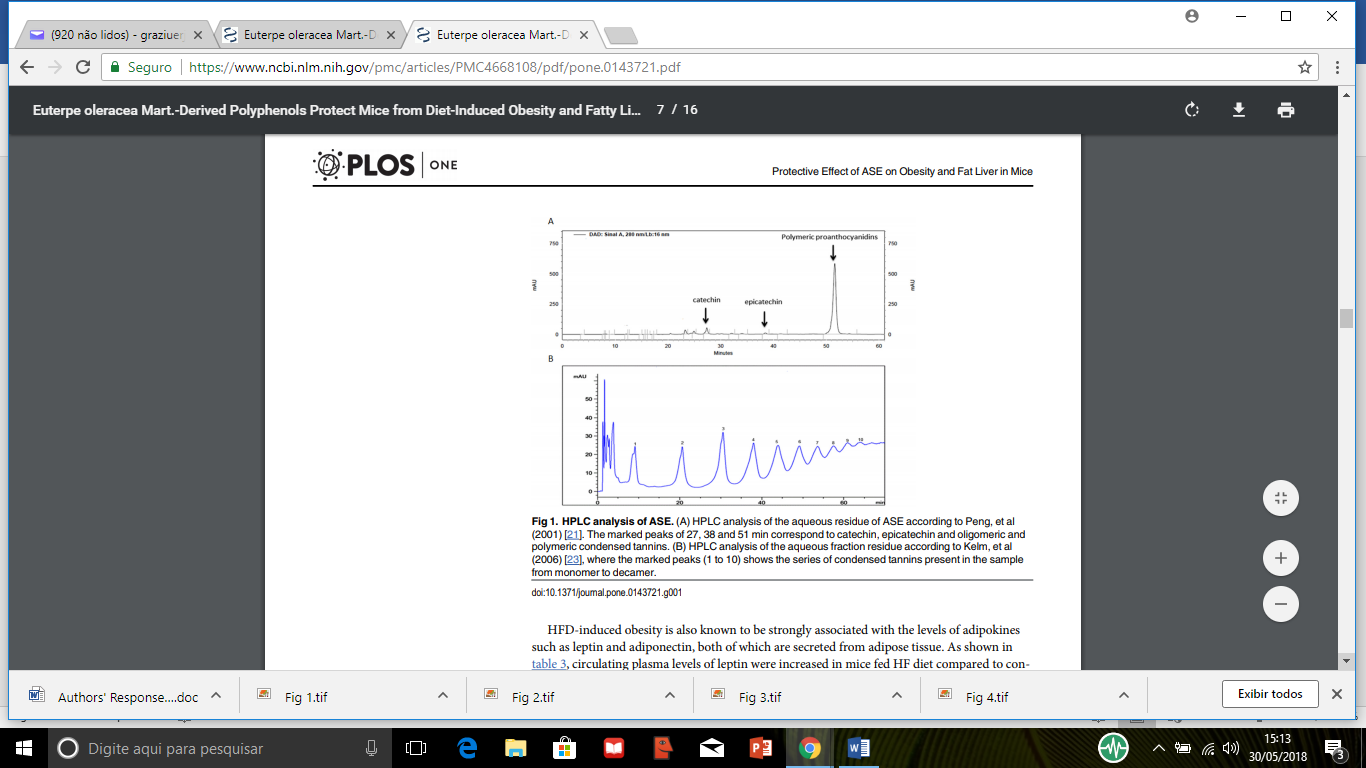


**Fig 1. HPLC analysis of ASE**

(A) HPLC analysis of the aqueous residue of ASE according to Peng, et al (2001). The marked peaks of 27, 38 and 51 min correspond to catechin, epicatechin and oligomeric and polymeric condensed tannins. (B) HPLC analysis of the aqueous fraction residue according to Kelm, et al (2006), where the marked peaks (1 to 10) shows the series of condensed tannins present in the sample from monomer to decamer. (Oliveira et al., PLOS ONE. 2015; 10: e0143721).


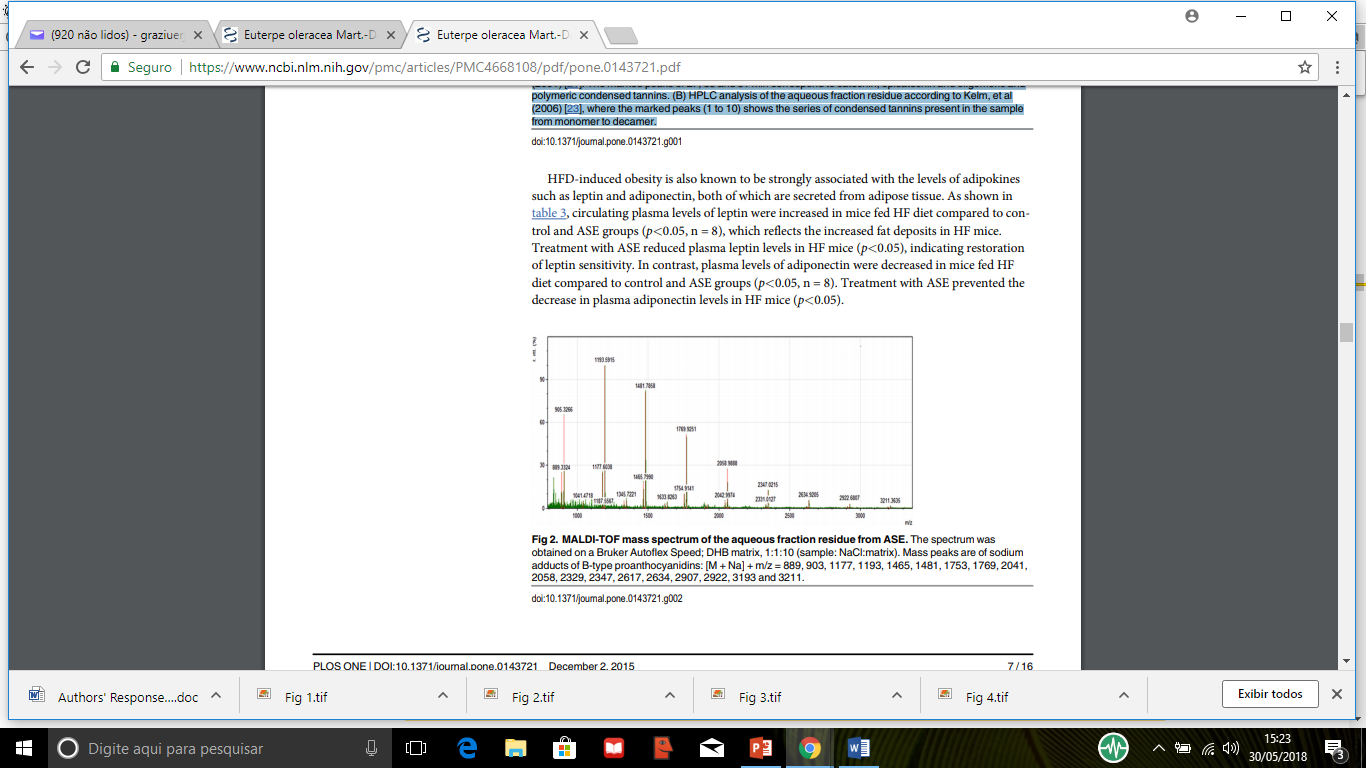


**Fig 2. MALDI-TOF mass spectrum of the aqueous fraction residue from ASE**

The spectrum was obtained on a Bruker Autoflex Speed; DHB matrix, 1:1:10 (sample: NaCl:matrix). Mass peaks are sodium adducts of B-type proanthocyanidins: [M + Na] + m/z = 889, 903, 1177, 1193, 1465, 1481, 1753, 1769, 2041, 2058, 2329, 2347, 2617, 2634, 2907, 2922, 3193 and 3211 (Oliveira et al., PLOS ONE. 2015; 10: e0143721).
